# Improving early diagnosis of rare diseases using Natural Language Processing in unstructured medical records: an illustration from Dravet syndrome

**DOI:** 10.1186/s13023-021-01936-9

**Published:** 2021-07-13

**Authors:** Tommaso Lo Barco, Mathieu Kuchenbuch, Nicolas Garcelon, Antoine Neuraz, Rima Nabbout

**Affiliations:** 1 Department of Pediatric Neurology, Necker-Enfants Malades Hospital, APHP, Centre de Référence Épilepsies Rares, Member of ERN EPICARE, Université de Paris, Paris, France; 2 Child Neuropsychiatry, Department of Surgical Sciences, Dentistry, Gynecology and Pediatrics, University of Verona, Verona, Italy; 3 Imagine Institute, INSERM, UMR 1163, Université de Paris, 75015 Paris, France; 4 Université de Paris, Paris, France; 5 INSERM, UMR1138, Centre de Recherche Des Cordeliers, Paris, France; 6 Department of Medical Informatics, University Hospital Necker-Enfants Malades, APHP, Paris, France

**Keywords:** Data mining, Natural Language Processing, Dravet syndrome, Rare Diseases, Early diagnosis

## Abstract

**Background:**

The growing use of Electronic Health Records (EHRs) is promoting the application of data mining in health-care. A promising use of big data in this field is to develop models to support early diagnosis and to establish natural history. Dravet Syndrome (DS) is a rare developmental and epileptic encephalopathy that commonly initiates in the first year of life with febrile seizures (FS). Age at diagnosis is often delayed after 2 years, as it is difficult to differentiate DS at onset from FS. We aimed to explore if some clinical terms (concepts) are significantly more used in the electronic narrative medical reports of individuals with DS before the age of 2 years compared to those of individuals with FS. These concepts would allow an earlier detection of patients with DS resulting in an earlier orientation toward expert centers that can provide early diagnosis and care.

**Methods:**

Data were collected from the *Necker Enfants Malades Hospital* using a document-based data warehouse, *Dr Warehouse,* which employs Natural Language Processing, a computer technology consisting in processing written information. Using Unified Medical Language System Meta-thesaurus, phenotype concepts can be recognized in medical reports. We selected individuals with DS (DS Cohort) and individuals with FS (FS Cohort) with confirmed diagnosis after the age of 4 years. A phenome-wide analysis was performed evaluating the statistical associations between the phenotypes of DS and FS, based on concepts found in the reports produced before 2 years and using a series of logistic regressions.

**Results:**

We found significative higher representation of concepts related to seizures’ phenotypes distinguishing DS from FS in the first phases, namely the major recurrence of complex febrile convulsions (long-lasting and/or with focal signs) and other seizure-types. Some typical early onset non-seizure concepts also emerged, in relation to neurodevelopment and gait disorders.

**Conclusions:**

Narrative medical reports of individuals younger than 2 years with FS contain specific concepts linked to DS diagnosis, which can be automatically detected by software exploiting NLP. This approach could represent an innovative and sustainable methodology to decrease time of diagnosis of DS and could be transposed to other rare diseases.

## Objectives

Electronic health records (EHRs) contain healthcare data of individuals and population electronically-stored in a digital format [1]. In the last decade, the use of EHRs has become part of routine care across the majority of developed countries [2].

Through data mining techniques, this growing use of EHRs is allowing the development of predictive models aimed to individuate high risk patients and support prevention initiatives [3, 4]. As well, models to support diagnosis and treatment of rare diseases are emerging [5, 6].

EHRs consist of structured and unstructured data. Structured data are produced through constrained choices (drop-down menus, check boxes and pre-filled templates as in registries), whereas unstructured clinical data exist in the form of free text narratives and are often used in clinical care for medical reports [7]. Combining Natural Language Processing (NLP) technology and UMLS (Unified Medical Language System), providers’ notes and narratives can be converted into structured, standardized formats, usable for data mining [8–10].

Dravet Syndrome (DS) is a rare disorder, with a worldwide incidence between 1/40,000 and 1/15,700 [11]. DS is a genetic developmental and epileptic encephalopathy with onset in first year of life, characterized at onset by febrile seizures and convulsive status epilepticus in otherwise healthy infants [12]. Starting by the second year, individuals present multiple seizure types (clonic, tonic–clonic, motor and non-motor onset focal seizures, myoclonic, atypical absences), that are often drug resistant, with developmental slowing leading to definite cognitive impairment [13]. Diagnosis is easier after the age of two as more pathognomonic seizure types and other symptoms are present from this age. Genetic testing shows a pathogenic variant in SCN1A in over 85% of cases reinforcing the diagnosis suspicion, but this testing might take months and is not available for all individuals with suspected DS [14]. However there is a need for early diagnosis in order to avoid worsening therapies and to establish best therapy protocol as seizure control might be partly related to cognitive improvement and a better quality of life [15].

Early diagnosis of individuals with DS is often delayed as it is difficult to differentiate at onset from Febrile Seizures (FS) [16]. These two conditions present substantial clinical differences, leading to exclude one on other diagnosis but might be overlapping at onset. Even if physician awareness of Dravet syndrome has markedly improved in last decades [17], time to diagnosis is still over 2 years [18], and it remains underdiagnosed in adult population and in developing countries [19, 20].

Using data mining, we analysed clinical reports produced before the age of 2 years for individuals with confirmed DS and FS with the aim of identifying specific terms (concepts) allowing early DS suspicion and reducing diagnosis delay. We then explored the differences between the concepts in the reports of two subgroups of individuals with DS: patients with suspected diagnosis before the age of 2 years and patients for whom diagnosis was suspected after the age of two.

## Materials and methods

Data were collected from *Necker Enfants Malades Hospital*, a paediatric University hospital belonging to the Assistance Publique Hopitaux de Paris group (400 paediatric beds, 200 adult beds), which is a national and European reference center for rare and undiagnosed diseases, including the reference a centre for rare epilepsies.

DrWarehouse® [21] (DrWH) is a document-based open-source data warehouse oriented toward narrative clinical reports from the Electronic health records (EHRs). It contains more than 4.5 million clinical free-text documents produced at Necker Hospital from 2009, for more than 465,000 individuals and more than 20 departments. DrWarehouse® uses UMLS Metathesaurus to recognize phenotype concepts inside narrative medical reports. In this manuscript, the word “concepts” will refer to phenotypes extracted automatically from hospital reports, without a priori, by using a UMLS subset of 20,000 phenotypic words or expressions.

By using the appropriate research field in DrWarehouse®, we searched all individuals who presented in their medical reports the word “Dravet” or “Severe Myoclonic Epilepsy of Infancy” at least in one clinical document. We then selected from this group all individuals that had a definite diagnosis of DS based on clinical and genetic criteria, and evaluated after the age of four where the full blown syndrome can be confirmed. We finally included from this group individuals with at least one clinical report before the age of 2 years and this final selection constituted the “Dravet Syndrome Cohort” (DS Cohort).

**Fig. 1:**
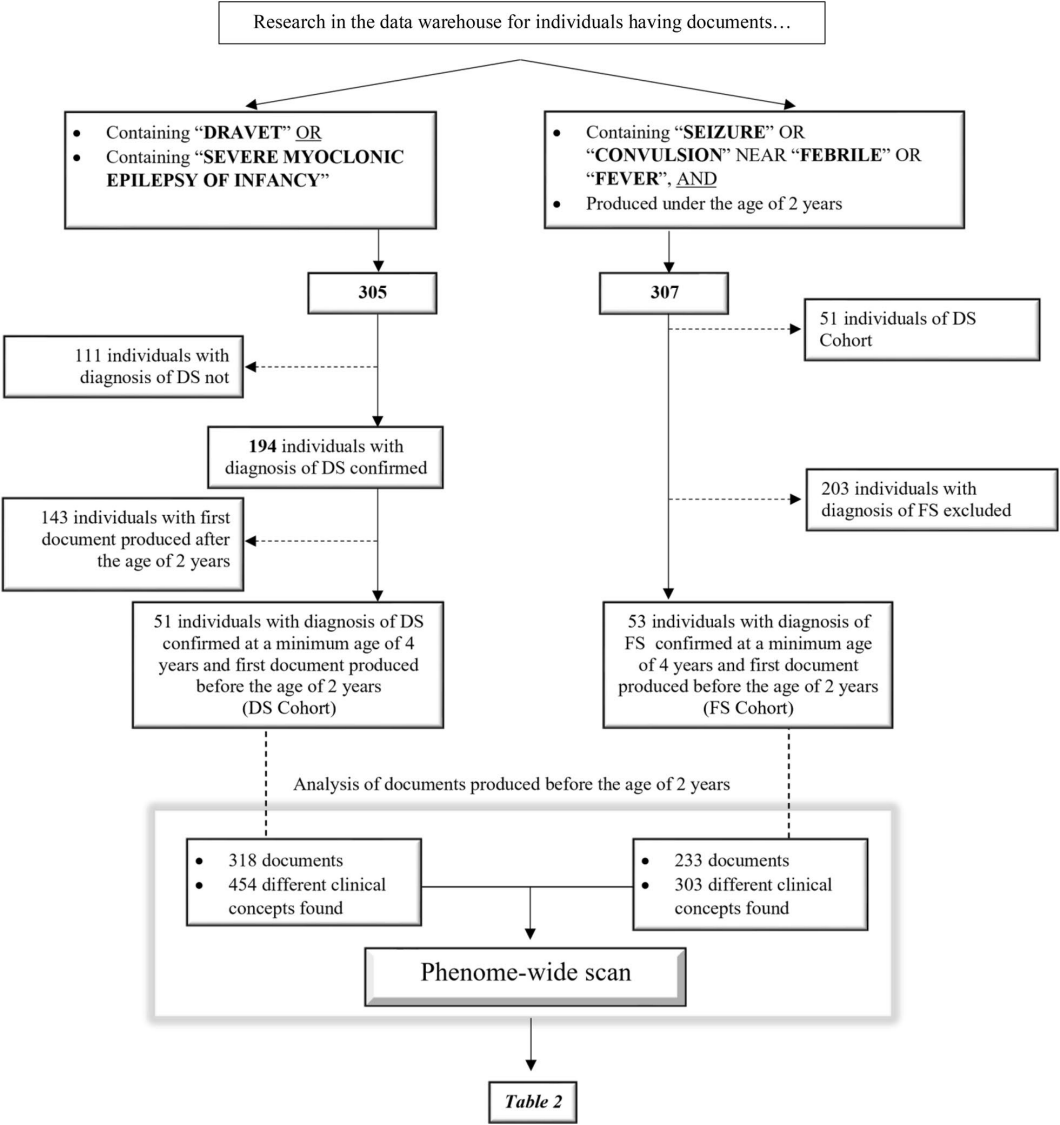
Flowchart of the selection procedures and constitution of the cohorts

Subsequently, we searched in the data of DrWarehouse all individuals whose medical reports produced before the age of two presented the words “seizure”/“seizures” or “convulsion”/“convulsions” in proximity (max 5 words away) to “fever” or “febrile”. From this group, we excluded the individuals of the DS Cohort and individuals in which febrile seizures was a symptom of a more complex condition (infections involving the central nervous system, other encephalopathies, structural brain injury, detected genetic or metabolic pathologies, or epilepsies). The “Febrile Seizures’cohort” (FS cohort) included the individuals from this group aged over year where we confirmed the diagnosis of febrile seizures based on EHRs or by telephone interviewing of the family (FS Cohort) (Fig. 1).

The phenome-wide scan consists in comparing the distribution of phenotypes between two groups (cases and controls) and estimates the association between the phenotypes and the groups. These associations are assessed sequentially [22, 23]. We evaluated the statistical associations between the phenotypes and the cohorts DS and FS, using a series of multivariate logistic regressions adjusted on gender and age. For the analysis, we used concepts found in clinical reports with a minimum number of occurrences of three individuals, excluding negations and those associated to family members. The *p-values* were corrected for multiple testing using a false discovery rate (FDR) methodology.

We also compared the phenotype differences in the DS Cohort between the subgroup where diagnosis of DS was confirmed or suspected before the age of 2 years, and the subgroup where DS diagnosis was not reported.

## Results

### “Dravet Syndrome Cohort” (DS Cohort)

The term “Dravet” and/or “severe myoclonic epilepsy of infancy” appeared in 305 individuals present in the warehouse: 194 of them had a final diagnosis of DS in the last document on the database, 51 had at least one document produced under the age of 2 years. All had a clinical and genetic diagnosis of DS. These individuals constituted the DS Cohort.

DS cohort included 28 males and 23 females. The mean age at first seizure was 5.5 months (min 2–max 12). The average age of the first produced document was 1.05 years, median is 1.15 (min 0.25–max 1.98). The average length of the follow-up of these individuals was 5.68 years, median 4.98 (min 3.75–max 13.42).

In order to compare early characteristics of this population with a population with FS at the same age, documents produced exclusively before 2 years were selected, for a total of 318 documents (mean: 6.24; median: 3 for each individual). 3484 concepts were extracted from the abovementioned documents (mean: 10.9 per document), 454 of which were unique concepts. Concepts present in almost 10% of the population are listed in a decreasing order in the Table 1. The most prevalent concepts were “Seizures” (found in 48 individuals – 94%), “Fever” (43 individuals – 84%), “Epilepsy” (42 individuals – 82%), “Dravet Syndrome” (37 individuals – 73%), “Convulsions” (31 individuals – 61%).

**Table 1:** Comparison between concepts found in more than 10% of individuals of DS Cohort (left) and FS Cohort (right)

UMLS CUI code	Concept	DS cohort		FS cohort	
		Number of individuals	Frequency (%)	Number of individuals	Frequency (%)
C0036572	Seizures	48	94	44	83
C0015967	Fever	43	84	48	91
C0014544	Epilepsy	42	82	35	66
C0751122	Dravet syndrome	37	73		
C0234972	Convulsions	31	61	40	75
C1419856	SCN1A	28	55		
C0009952	Febrile seizures	21	41	37	70
C0026827	Hypotonia	21	41	19	36
C2825055	Recurrence	21	41	30	57
C0234535	Clonic	17	33	9	17
C1705236	Deviation	17	33		
C3809175	Prolonged seizure	16	31	8	15
C2830004	Drowsiness	16	31	17	32
C0038220	Status epilepticus	15	29	8	15
C0259972	Ketogenic diet	13	25		
C0235195	Sedation	13	25		
C3887612	Psychomotor agitation	12	24	7	13
C0009450	Infection	12	24	9	17
C0027066	Myoclonia	11	22		
C1698630	Virosis	11	22	11	21
C0030193	Pain	10	20	6	11
C0013144	Falling asleep	10	20	12	23
C0026837	Hypertonus	10	20	8	15
C0029877	Otitis	10	20	13	25
C0522336	Rolling of eyes	10	20	18	34
C0010200	Cough	10	20	9	17
C0004134	Ataxia	9	18		
C0006271	Bronchiolitis	9	18	11	21
C0009443	Rhinitis	9	18	14	26
C0085639	Fall	8	16		
C0424927	Education	8	16		
C0024115	Pneumonia	8	16		
C0684320	Regression	8	16		
C0020517	Allergies	7	14		
C0751495	Focal Seizures	7	14		
C0015672	Fatigue	7	14	7	13
C0026205	Myosis	7	14		
C0036973	Shiverings	7	14		
C0035561	Side	6	12		
C0031350	Pharingitis	6	12	8	15
C0424230	Psychomotor DELAY	6	12		
C0027441	Nasopharyngitis	6	12		
C0549209	Startle	6	12		
C0003123	Anorexia	5	10		
C0034642	Rales	5	10		
C0270844	Tonic Seizures	5	10	6	11
C0010520	Cyanosis	5	10	9	17
C0011991	Diarrhea	5	10		
C0017160	Gastroenteritis	5	10		
C0019209	Hepatomegaly	5	10		
C0013384	Movement disorder	5	10		
C0013604	Edema	5	10		
C0232483	Reflux	5	10		
C0035203	Respiration	5	10		
C0037763	Spasms	5	10		
C1504405	Pyramidal syndrome	5	10		
C0008049	Chickenpox	5	10	7	13
C0596002	Reflex			19	36
C0271429	Acute otitis media			9	17
C0855324	Normal pulse pressure			8	15
C0042963	Vomiting			8	15
C0034150	Purpura			7	13
C0494475	Tonic–clonic seizures			6	11

*CUI* concept unique identifiers

### “Febrile Seizure Cohort” (FS Cohort)

The research of the words “seizure” or “convulsion” in individuals’ reports close to the words “febrile” or “fever”, limited to documents produced by the first 2 years of life and excluding individuals of DS Cohort, led to 256 subjects. After exclusion of other aetiologies, we included all 53 subjects with a diagnosis of febrile seizures. Diagnosis was confirmed after age four by reviewing child's medical history, neurological and developmental outcome in the available medical files in addition to a telephone interview with the family.

This cohort was constituted of 17 females and 36 males. The mean age of the first document produced was 1.18 years, while median was 1.3 (min 0.30–max 1.96). The mean duration of follow-up was 4.20 years, median 4.02 (min 3.70–max 5.57). The mean age at first seizure was 12.4 months (min 4–max 21) with 1 individual having an onset before 6 months and 23 before 12 months.

In order to compare phenotypes of FS Cohort with DS Cohort at the same age (before the age of 2 years), documents produced exclusively before 2 years were selected, for a total of 233 documents (mean 4.4; median 3 for each individual). From these, 2053 concepts have been extrapolated (mean 8.8 concepts per document), 303 of which were unique concepts.

The concepts present in more than 10% of individuals are shown in Table 1. The most prevalent concepts were “Fever” (found in 48 individuals—91%), “Seizures” (44 individuals—83%), “Convulsions” (40 individuals—75%), “Febrile Seizures” (37 individuals—70%), “Epilepsy” (35 individuals—66%).

### Comparison of DS and FS cohorts

DS cohort was constituted of 54% of males and 46% of females while in FS cohort, gender comparison showed significant difference with 68% of males and 32% of females (*p* = 0.009).

The different length of follow-up at our centre among the two cohorts shows the higher medical needs for individuals with DS (mean 3.99 years, median 3.11) compared to individuals with FS (mean 1.82 years, median 1.37 years). Indeed, the follow-up at our centre often stops when the diagnosis of FS is confirmed, and children are usually referred back to their paediatrician or general practitioner.

The mean number of documents per individual produced during the same period (0–2 years), was higher in the population with DS (6.2 vs 4.4), as well as the mean number of concepts extrapolated per document (10.9 vs 8.8).

The phenome-wide comparison of both cohorts showed a different representation of a series of concepts (Table 2). Some of these concepts were related to seizures. Concept “Deviation” (*p* < 0.01), which is found within sentences describing focal seizures, point out to a significant higher occurrence of focal seizures in DS cohort compared to FS cohort. The frequency of “prolonged seizures” concept was also significantly higher in DS cohort (31% compared to 15% in FS cohort, *p* = 0.05*.* Another concept, “sedation”, which was used in the medical reports with reference to the post-ictal phase or to the need of rescue medication showed a significant difference (25% in the DS Cohort, 0% in the FS Cohort; *p* = 0.02)*.* The concept “myoclonia” was not found in the FS Cohort, while was reported in 22% of individuals of DS Cohort (*p* = 0.02), and the concept “clonic” was reported two folds in the DS Cohort compared to the FS one (33% versus 17%, *p* = 0.05). The concept “febrile seizures” was significantly higher in the FS Cohort and was found in 70% of individuals compared to 41% of individuals of DS Cohort (*p* = 0.01). Other non seizures concepts were found only in the DS Cohort, namely “ataxia” (18%; *p* = 0.02)*,* “regression” (16%; *p* = 0.03) and “pneumonia” (16%; *p* = 0.03).

**Table 2:** Phenome-wide comparison of DS Cohort and FS Cohort

UMLS CUI code	Concept	DS individuals with the concept(%)	FS individuals with the concept (%)	DS individuals without the concept (%)	FS individuals without the concept (%)	OR	*p* value
C0751122	Dravet syndrome	36 (70.6)	0 (0)	15 (29.4)	53 (100)	129.60	0.00
C1419856	SCN1A	28 (54.9)	0 (0)	23 (45.1)	53 (100)	65.74	0.00
C1705236	Deviation	17 (33.3)	4 (7.5)	34 (66.7)	49 (92.5)	6.37	0.00
C0259972	Ketogenic diet	13 (25.5)	0 (0)	38 (74.5)	53 (100)	18.47	0.01
C0009952	Febrile seizures	21 (41.2)	37 (69.8)	30 (58.8)	16 (30.2)	0.34	0.01
C0235195	Sedation	13 (25.5)	4 (7.5)	38 (74.5)	49 (92.5)	4.36	0.02
C0027066	Myoclonia	11 (21.6)	3 (5.7)	40 (78.4)	50 (94.3)	4.77	0.02
C0004134	Ataxia	9 (17.6)	1 (1.9)	42 (82.4)	52 (98.1)	11.57	0.02
C0024115	Pneumonia	8 (15.7)	0 (0)	43 (84.3)	53 (100)	10.05	0.03
C0684320	Regression	8 (15.7)	0 (0)	43 (84.3)	53 (100)	10.05	0.03
C0234535	Clonic	17 (33.3)	9 (17)	34 (66.7)	44 (83)	2.56	0.05
C0085639	Fall	8 (15.7)	2 (3.8)	43 (84.3)	51 (96.2)	4.93	0.05
C3809175	Prolonged seizure	16 (31.4)	8 (15.1)	35 (29.6)	45 (84.9)	2.87	0.05
C0014544	Epilepsy	41 (80.4)	35 (66)	10 (19.6)	18 (34)	2.34	0.06
C0038220	Status epilepticus	15 (29.4)	8 (15.1)	36 (70.6)	45 (84.9)	2.45	0.07
C0424230	Psychomotor delay	6 (11.8)	0 (0)	45 (88.2)	53 (100)	7.20	0.07
C0549209	Startle	6 (11.8)	0 (0)	45 (88.2)	53 (100)	7.20	0.07
C0855324	Normal pulse pressure	2 (3.9)	8 (15.1)	49 (96.1)	52 (98.1)	0.24	0.08
C0026205	Myosis	7 (13.7)	2 (3.8)	44 (86.3)	51 (96.2)	4.22	0.08
C0036572	Seizures	47 (92.9)	44 (83)	4 (7.8)	9 (17)	2.94	0.08
C0271429	Acute otitis media	3 (5.9)	9 (17)	48 (94.1)	44 (83)	0.32	0.10
C0003123	Anorexia	5 (9.8)	0 (0)	46 (90.2)	53 (100)	5.87	0.11
C0013604	Edema	5 (9.8)	1 (1.9)	46 (90.2)	52 (98.1)	5.87	0.11
C0035203	Respiration	5 (9.8)	1 (1.9)	46 (90.2)	52 (98.1)	5.87	0.11
C0037763	Spasms	5 (9.8)	1 (1.9)	46 (90.2)	52 (98.1)	5.87	0.11
C0034150	Purpura	2 (3.9)	7 (13.2)	49 (96.1)	46 (86.8)	0.28	0.12
C0522336	Rolling of eyes	10 (19.6)	18 (34)	41 (80.4)	35 (66)	0.50	0.13
C0231218	Malaise	1 (2)	5 (9.4)	50 (98)	48 (90.6)	0.20	0.15
C0427008	Stiffness	1 (2)	5 (9.4)	50 (98)	48 (90.6)	0.20	0.15
C1336751	Flat	1 (2)	5 (9.4)	50 (98)	48 (90.6)	0.20	0.15
C3887612	Pyschomotor agitation	12 (23.5)	7 (13.2)	39 (76.5)	46 (86.8)	2.11	0.15
C0042963	Vomiting	3 (5.9)	8 (15.1)	48 (94.1)	45 (84.9)	0.37	0.16
C0036973	Shiverings	7 (13.7)	3 (5.7)	44 (86.3)	50 (94.3)	2.76	0.16
C0751495	Focal seizures	7 (13.7)	3 (5.7)	44 (86.3)	50 (94.3)	2.76	0.16
C2825055	Recurrence	21 (41.2)	30 (56.6)	30 (58.8)	23 (43.4)	0.58	0.17
C0013473	Eating disorders	4 (7.8)	0 (0)	47 (92.9)	53 (100)	4.60	0.18
C0018989	Hemiparesis	4 (7.8)	1 (1.9)	47 (92.9)	52 (98.1)	4.60	0.18
C0020649	Hypotension	4 (7.8)	0 (0)	47 (92.9)	53 (100)	4.60	0.18
C0032290	Aspiration Pneumonia	4 (7.8)	0 (0)	47 (92.9)	53 (100)	4.60	0.18
C0037036	Hypersalivation	4 (7.8)	0 (0)	47 (92.9)	53 (100)	4.60	0.18
C0038450	Stridor	4 (7.8)	0 (0)	47 (92.9)	53 (100)	4.60	0.18
C0205721	Nosocomial infection	4 (7.8)	1 (1.9)	47 (92.9)	52 (98.1)	4.60	0.18
C0333641	Atrophy	4 (7.8)	0 (0)	47 (92.9)	53 (100)	4.60	0.18
C0349506	Photosensitivity	4 (7.8)	0 (0)	47 (92.9)	53 (100)	4.60	0.18
C0428167	FiO2	4 (7.8)	1 (1.9)	47 (92.9)	52 (98.1)	4.60	0.18
C0865850	Acute respiratory insufficiency	4 (7.8)	0 (0)	47 (92.9)	53 (100)	4.60	0.18
C1504405	Pyramidal syndrome	4 (7.8)	0 (0)	47 (92.9)	53 (100)	4.60	0.18

*CUI* concept unique identifiers

In addition, a series of concepts were consistently more represented in the DS Cohort than in FS Cohort, without reaching a statistical significance as “status epilepticus” (29% versus 15%; *p* = 0.07, *OR* = 2.4), “startle” (12 versus 0%; *p* = 0.07, *OR* = 7.2)*,* “psychomotor delay” (12 versus 0%; *p* = 0.07, *OR* = 7.2)*,* “pyramidal syndrome” (10 *versus* 0%; *p* = 0.18, *OR* = 4.6) *“*hemiparesis” (8 versus 2%; *p* = 0.18, *OR* = 4.6) and “photosensitivity” (8 *versus* 0%; *p* = 0.18, *OR* = 4.6).

### Analysis of the DS cohort in regard to the early diagnosis

In the DS cohort, we compared the subgroup of individuals who had DS diagnosis confirmed or suspected before the age of 2 years of age (n = 36) *versus* the subgroup where the diagnosis of DS was not suspected (n = 15). In the first, the term (concept) Dravet syndrome was reported in the clinical reports before the age of 2 years while none of the individuals of the second group had any use of this term suggesting that DS diagnosis was not suspected before the age of 2 years. The mean age at first seizure was 5.3 months (min 2–max 12) in the subpopulation that received a diagnosis or a suspected diagnosis before age 2 and 6.1 months (min 2 – max 9) in the group without an early diagnosis (*p* = 0.2). Individuals who received diagnosis within 2 years showed a higher rate of concepts as “seizures” (*p* < 0.01), “fever” (*p* < 0.01), “epilepsy” (*p* < 0.01), “prolonged seizures” (*p* < 0.01), “convulsions” (*p* = 0.01), “myoclonia” (*p* = 0.02) and “ataxia” (*p* = 0.04) compared to the second group (Table 3).

**Table 3:** Comparison between concepts found in more than 10% individuals of DS Cohort who received the diagnosis/suspicion of DS before (left) and after (right) the age of 2 years

UMLS CUI code	Concept	DS cohort diag < 2 years		DS cohort diag > 2 years	
		Number of individuals	Frequency (%)	Number of individuals	Frequency (%)
C0751122	Dravet syndrome	36	100		
C0036572	Seizures	36	100	11	79
C0015967	Fever	34	94	8	57
C0014544	Epilepsy	33	92	8	57
C0234972	Convulsions	26	72	5	36
C1419856	SCN1A	22	61	6	43
C0026827	Hypotonia	17	47	4	29
C2825055	Recurrence	17	47	4	29
C0009952	Febrile seizures	15	42	5	36
C3809175	Prolonged seizure	15	42		
C0234535	Clonic	14	39	3	21
C0038220	Status epilepticus	13	36	2	14
C0596002	Reflex	13	36	2	14
C1705236	Deviation	12	33	5	36
C2830004	Drowsiness	12	33	4	29
C0259972	Ketogenic diet	11	31	2	14
C0235195	Sedation	11	31	2	14
C1698630	Virosis	11	31		
C3887612	Psychomotor Agitation	10	28	2	14
C0009450	Infection	10	28		
C0027066	Myoclonia	10	28		
C0004134	Ataxia	9	25		
C0030193	Pain	9	25		
C0013144	Falling asleep	9	25		
C0029877	Otitis	9	25		
C0009443	Rhinitis	9	25		
C0010200	Cough	9	25		
C0424927	Education	8	22		
C0522336	Rolling of eyes	8	22	2	14
C0020517	Allergies	7	19		
C0006271	Bronchiolitis	7	19	2	14
C0015672	Fatigue	7	19		
C0026837	Hypertonus	7	19	3	21
C0026205	Myosis	7	19		
C0024115	Pneumonia	7	19		
C0085639	Fall	6	17		
C0494475	Tonic–clonic seizures	6	17		
C0751495	Focal seizures	6	17		
C0031350	Pharingitis	6	17		
C0424230	Psychomotor delay	6	17		
C0684320	Regression	6	17	2	14
C0549209	Startle	6	17		
C0036973	Shiverings	6	17		
C0003123	Anorexia	5	14		
C0035561	Side	5	14		
C0034642	Rales	5	14		
C0013604	Edema	5	14		
C0035203	Respiration	5	14		
C0424230	Psychomotor delay	5	14		
C0008049	Chickenpox	5	14		
C0004093	Asthenia	4	11		
C0270844	Tonic seizures	4	11		
C0010520	Cyanosis	4	11		
C0011991	Diarrhea	4	11		
C0221725	Bronchial obstruction	4	11		
C0017160	Gastroenteritis	4	11		
C0021400	Flu	4	11		
C0037036	Hypersalivation	4	11		
C0042769	Viral infection	4	11		
C0349506	Photosensitivity	4	11		
C0032290	Aspiration pneumonia	4	11		
C1272641	Arterial blood pressure	4	11		
C0027441	Nasopharyngitis	4	11	2	14
C0037763	Spasms	4	11		
C0038450	Stridor	4	11		
C1504405	Pyramidal syndrome	4	11		
C0018916	Angiome			2	14
C0085584	Encephalopathy			2	14
C0018989	Hemiparesis			2	14
C0018991	Hemiplegia			2	14
C0019209	Hepatomegaly			2	14
C0232483	Reflux			2	14

*CUI* concept unique identifiers

## Discussion

This study shows that narrative medical reports produced before 2 years include several clinical concepts which are significantly associated with individuals with DS compared to FS, this latter condition representing the main differential diagnosis at the onset. These concepts are consistent with the main clinical findings constituting the criteria for differentiating DS from FS in first 2 years of life.

FS are usually reported after the first year with some cases initiating before 12 months. They are usually brief and generalized [24]. In our study, concepts referred to prolonged (“status epilepticus”, “prolonged seizures”, “sedation”) and focal seizures (“deviation”) are prominent in the DS cohort, emphasizing the higher tendency of individuals with DS to present at onset long lasting and focal febrile seizures compared to individuals with FS [16, 25, 26]. Importantly, individuals with DS develop different types of seizures as myoclonic or atypical absences in addition to the first seizures mimicking FS. We observed in our DS cohort concepts referring to seizures other than febrile convulsions, including “Myoclonia” and “startle”, which is mostly used in narrative reports to depict myoclonic seizure semiology [16, 27, 28]. The concept “hemiparesis” was more frequent in the DS Cohort compared to FS one. This is consistent with the higher occurrence of transitory hemiplegia after long-lasting hemiclonic seizures, a type of seizure being quite suggestive of DS [16, 27, 29].

Some important non-seizure concepts also emerged, differentiating the two cohorts. Subjects with DS and FS show a normal neurodevelopment at the seizure onset, but then psychomotor trajectories deviate [26, 30]. In accordance, concepts related to psychomotor delay were found only in the DS Cohort (“Regression”, “Psychomotor delay”). In addition, “Ataxia” was significantly more reported DS Cohort, reflecting the peculiar gait disorder commonly observed in individuals with DS, and representing an early motor-marker of this condition [28, 31].

Interestingly, the concept “febrile seizures” was found with significant higher frequency in the FS Cohort probably because it was used for a “diagnostic” purpose in the clinical reports.

The study was carried out in a tertiary epilepsy center, so it is plausible that some words have been chosen as a consequence of the clinical suspicion of Dravet Syndrome by highly experienced specialist in epileptology (e.g. “myoclonia”, “ataxia”). However, many of the medical reports were done by physicians without a specific expertise in epilepsy or DS (e.g. emergency care or intensive care physicians), emphasizing the uniformity of expressions used for reporting disease and individuals description, and suggesting that most of key-concepts may have also been found into non-specialists medical reports (e.g. “deviation”, “prolonged seizures”, “startle”).

Several studies show a substantial worldwide issue of diagnostic delay of DS, with a mean age at diagnosis that is usually over 2 years, resulting in “unnecessary, costly, and, at times, invasive testing, and use of ineffective therapies, which can exacerbate seizures, increase the risk of status epilepticus, and worsen cognitive outcome” [17, 32–34]. Moreover, DS is certainly less recognized in adult population and in developing countries [19, 20].

Computer-based models using EHRs able to suggest diagnosis and to avoid misdiagnosis are gaining ground [3, 35]. These models are mostly based on structured data, as image-based or laboratory data [36, 37]. Recently, more complex models of artificial intelligence are emerging, which are able to elaborate diagnosis by extracting clinically relevant information from unstructured data in EHRs [38, 39].

On the basis of our findings, further extensive studies might focus on elaborating a specific computer algorithm which combines significative concepts and their age of appearance within narrative specialists and non-specialists reports, in order to automatically produce an alert signal suggesting possible diagnosis of DS.

Some results of our analysis set out some additional insights. For example, the major incidence of concept “pneumonia” in DS Cohort compared to FS Cohort appears to be relevant, since it can represent both a facilitator of the seizure onset or  a complication of an inhalation during a long lasting convulsive seizure or a status epilepticus [40]. In addition, a number of concepts related to peri-ictal nosocomial and respiratory complications were found with higher frequency in reports of individuals with DS (“nosocomial infections”, “acute respiratory insufficiency”, “aspiration pneumonia”, “FiO2”, “stridor”) underlying that convulsive status epilepticus might be a life-threatening condition in this population [40, 41].

Furthermore, in this study the concept “Dravet Syndrome” was found in 72% of individuals of DS Cohort before the age of 2 years. This is concordant with the literature showing the early recognition of DS in France [34].

Some clinical concepts were found with higher frequency in the reports of individuals who received the diagnosis/suspicion of DS before the age of 2 years: the “long-lasting seizure” concepts (“Status epilepticus”, “Prolonged seizures”, “Sedation”), the “myoclonic” concepts (“Myoclonia”, “Startles”), the “drug resistance” concepts (“Ketogenic diet”), as well as “Ataxia”, and “Photosensitivity”. Although statistical significance was not reached for all these concepts as sample was small, these findings may support that these clinical concepts are the most DS diagnosis orienting. We can hypothesise that individuals belonging to the sub-group who did not receive a diagnosis within 2 years presented a less “typical” phenotype. The diagnosis was made later than 2 years of age when the full blown syndrome is often complete with pharmacoresistant seizures and developmental plateauing. However, in this subgroup without early diagnosis with individuals presenting “intermediate” features between only FS and the “complete” DS clinical picture, the median age at first seizure was significantly lower than in FS cohort (6.1 months vs 12.4 months). This finding confirms that age at first seizure might be the strongest predictor of DS in infants who experience febrile seizures [25].

### Study limitations

Word sense disambiguation poses a challenge in extracting meaningful data from unstructured text. Clinical notes often contain terms or phrases that have more than one meaning [8], or that need for a contextualisation to understand the real clinical meaning. For example, concept “deviation” apparently do not link to a specific clinical feature, but in the narrative reports of individuals of both cohorts it was mostly used within the description of the seizure semeiology, thus referring to a focal seizure.

The presence of a clinical concept in a medical report does not necessary implies that the individual presents this clinical feature. For instance, the concept “spasms” that we found in five individuals of the DS Cohort, was used within the clinical description of paroxysmal motor events that could suggest epileptic spasms, but was not confirmed in any of them. Similarly, concept “Dravet Syndrome” could be found in reports of subjects who received the diagnosis, or in which a suspicion was made (i.e.: “We see today patient X for the suspicion of Dravet Syndrome”)*.* The method used by Dr Warehouse automatically classifies concepts according to polarity (negation/affirmation) and the experiencer (patient/family). But there may still be errors in the classification. In addition, the classification does not take into account the notion of hypothesis.

In this study, the FS population presents some “atypical” features; for instance, the frequency of the concept “status epilepticus” in these subjects is higher than expected in terms of incidence in individuals with febrile seizures [42, 43]. This might be due to a preferential referral to university hospital of individuals with febrile long lasting seizures or febrile status epilepticus, as they might need further admission to ICU.

## Conclusion

Narrative medical reports of individuals younger than 2 years with febrile seizures, contain different words depending if they have or will develop clinical phenotype of DS, or not. The elaboration of algorithm exploiting NLP on the basis of our work, could be useful to early individualize these individuals, in order to establish early diagnosis and adequate therapy that in some instances need to address them to expert epilepsy centres.

This methodology would represent an innovative, “cheap”, transposable and sustainable methodology to reduce time of diagnosis for individuals with Dravet Syndrome and other rare conditions.

Some “key early symptoms” often identified by the patients/care givers and the non-expert physicians are merely linked to a given known disease causing diagnosis delay. Using these symptoms and signs as alerts and warning signs can help to address patients earlier to expert centres for a definite diagnosis. The future step is to validate the impact of the implementing of these “warnings” in the electronic health records on shortening the patient’s odyssey to diagnosis and therapies.

## Data Availability

The datasets used and analysed during the current study are available from the corresponding author on reasonable request.
